# The diagnosis of Adamantiades-Behçet disease: Clinical features and diagnostic/classification criteria

**DOI:** 10.3389/fmed.2022.1098351

**Published:** 2022-12-09

**Authors:** Serena Bergamo

**Affiliations:** Dermatology Unit, ULSS 2 Marca Trevigiana, Ca’ Foncello Hospital, Treviso, Italy

**Keywords:** Behçet, criteria, diagnosis, Adamantiades-Behçet disease, diagnostic criteria, microangiopathy

## Abstract

Adamantiades-Behçet’s disease (ABD) is a chronic-relapsing multisystemic inflammatory disease with unknown etiology first described by a Greek ophthalmology Benediktos Adamantiades and a Turkish dermatology Hulusi Behçet. Any organ or apparatus may be involved, though more often there is an involvement of oral and genital mucosae as well as ocular lesions, skin features, and vascular findings. Since there is neither laboratory nor radiological pathognomonic test, the diagnosis is basically clinical according to peculiar signs and symptoms of the disease. With the purpose of giving objectivity and homogeneity to the diagnosis, many authors in time introduced a long series of diagnostic and classification criteria for Adamantiades-Behçet’s disease. This mini-review provides an overview of published diagnostic/classification criteria.

## History

The very first description of the disease probably is to be referred neither to Adamantiades nor to Behçet, in fact Hippocrates of Kos (460-377 BC) wrote in the third *Epidemion* book about a condition with mouth ulcers, swelling in the genital area, watery inflammation of the eyes having a chronic course which strongly reminds of the peculiar clinical features of Adamantiades-Behçet disease. After the work of Hippocrates, no further description of the disease can be found in medical literature until the 20th Century.

In 1930, at the annual meeting of the Medical Society of Athens, an ophthalmologist named Benediktos Adamantiades presented a case of “relapsing iritis with hypopyon” correlating genital ulcers, arthritis, and ocular signs as part of a single disease (1).

Later in 1946, Adamantiades described two more cases with the addition of thrombophlebitis as the fourth sign of the disease (2). Few years later, he proposed the first classification of the condition describing the ocular, mucocutaneous, and systemic forms pointing out that ocular involvement and a more severe clinical course are more common in male patients (3). In 1958, Adamantiades published a manuscript about the neurological sequelae of the disease (4).

A Turkish dermatologist named Behçet, in 1937, at the Dermatology Association meeting in Istanbul presented a case of a 34-year-old female patient with recurrent oral aphthosis, genital ulcers, and ocular lesion. Later in the same year, a case of a 40-year-old patient with a 20-year history of the same disease was reported, speculating a viral etiology (5, 6). Later, Behçet defined a new “three symptom disease” with oral aphtosis, genital aphtosis, and ocular lesions as main signs, adding in the spectrum of the condition also periodontitis, maxillary cists, acneiform eruptions, erythema nodosum, and arthralgia (7).

The name Adamantiades-Behçet disease (ABD) honors both physicians who in the modern era recognized within its several clinical features a unique autonomous and not yet described disease (8, 9).

## Clinical signs of Adamantiades-Behçet’s disease

Adamantiades-Behçet disease is a systemic vasculitis characterized by a wide range of clinical features, presenting with mucocutaneous, ocular, vascular, neurological, rheumatological, and gastrointestinal manifestations, every organ or apparatus can be involved by the disease (10).

Oral aphtosis in ABD is a common feature, constituted by recurrent and painful, single or multiple oral ulcers usually located on lips, buccal mucosa, soft palate, and tongue. Genital ulcers often start as a papule or a pustule that develops a painful ulcer covered by fibrin. In male patients, they are usually located on the scrotum, penis, and glans, whereas in females they can be found in labia minora and majora, vagina, and more rarely in the cervix. Skin manifestations are common in ABD and are represented by papulo-pustolosis, erythema nodosum–like lesions, pseudofolliculitis, cutaneous ulcers, cellulitis-like lesions, Sweet syndrome, and cutaneous vasculitic lesions (9, 10).

**FIGURE 1:**
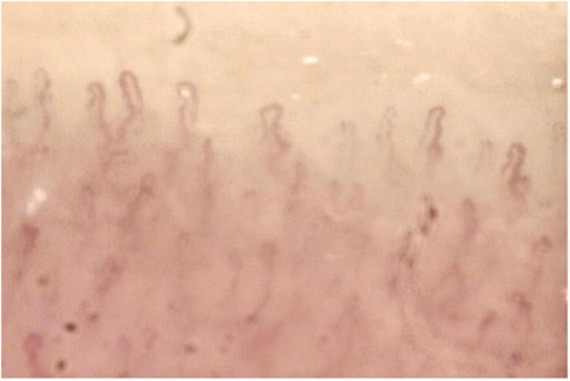
Capillaroscopy in a patient with Adamantiades-Behçet disease (ABD) and Raynaud’s phenomenon showing non-specific microangiopathy. Normal number of capillaries/mm, tortuous capillaries, and filiform capillaries with minor dystrophies, pericapillary edema.

Anterior and posterior uveitis, retinal vasculitis, scleritis, keratitis and keratoconjunctivitis, and optic neuritis are common clinical findings in ABD. Vascular involvement is common in ABD, affecting both arteries and veins. Arterial manifestations consist of arteritis, arterial thrombosis, and aneurism formation. Deep venous thrombosis and superficial phlebitis are signs of venous involvement. Raynaud’s phenomenon, though rare, can be found in patients with ABD. Capillaroscopy can be a useful tool in ABD patients with Raynaud’s phenomenon showing non-specific elements of microangiopathy such as non-parallel distributions of vessels and minor dystrophies (Figure 1). Raynaud’s phenomenon may be evoked in capillaroscopy through cold-pressure test (immersion of the fingers in cold water at 12°C for 3 min). Movasat and colleagues performed nail-fold capillaroscopy in 128 patients with ABD finding abnormalities such as enlarged capillaries and hemorrhages in 51 patients (11).

Arthralgia and arthritis are common in patients with ABD, affecting more commonly knees, ankles, elbows, wrists, and less frequently small joints of hands and feet. Neurological involvement can be acute or chronic and represents a severe feature of the disease being associated with elevated morbidity and mortality. Meningoencephalitis is the classical manifestation in ABD patients, and it is considered to depend on vasculitis of small vessels, mainly veins. Patients with ABD can complain of diarrhea, nausea, vomiting, and anorexia as an expression of gastrointestinal involvement. Aphthous ulcers can be found in any tract of the gastrointestinal system, more commonly in the terminal ileum and cecum, simulating inflammatory bowel disease.

Pulmonary involvement is rare though represents an important cause of death in ABD patients. The main features of lung ABD are pulmonary artery aneurysms, pulmonary artery thrombosis, and pulmonary thromboembolism associated with pulmonary artery vasculitis.

Pericarditis, endocarditis, alterations of coronary arteries such as aneurysms and stenosis, and myocarditis represent signs of cardiac involvement.

Sometimes, in asymptomatic patients, urine exam may show microhematuria and proteinuria, as signs of renal involvement in terms of focal glomerulonephritis.

Other features such as vesical-vaginal fistulae, orchitis and epididymitis, rhinosinusitis, and recurrent otitis can be found in patients with ABD: as a matter of fact, the condition can involve any organ or apparatus (9, 10).

## Diagnosis and classification of Adamantiades-Behçet disease

### Introduction

Adamantiades-Behçet disease is a chronic-relapsing multisystemic inflammatory disease with an unknown etiology (9). The wide spectrum of clinical features is one of its main characteristics. Clinically, the disease presents a large variety of mucocutaneous, ocular, vascular manifestation, as well as central nervous system, muscle-skeleton apparatus, and gastrointestinal tract abnormalities: any organ or apparatus may be involved (10).

Since neither pathognomonic symptoms nor laboratory or radiological tests are available, the diagnosis of ABD depends on criteria that have to contain the main clinical features of the condition.

### The criteria before 1990

With the purpose of providing objectivity to the diagnosis, many authors, since the first descriptions of the disease, introduced a long series of diagnostic and classification criteria for ABD.

Curth in 1946 was the first author who proposed a diagnostic criterion for the diagnosis of ABD, only a few years later the description of the condition (12), followed by Hewitt et al. (13). In 1969, Mason and Barnes were the first to suggest a division between major (oral and genital ulcers, ocular and cutaneous lesions) and minor symptoms (gastrointestinal lesions, thrombophlebitis, cardiovascular findings, arthritis, central nervous system lesions, and family history). According to Mason and Barnes, ABD was diagnosed when at least three major symptoms or two major and two minor symptoms were present (14). These criteria were followed by others by Hewett et al. (15), then in 1972 by the Japanese Committee (16), Hubault and Hamza (17), O’Duffy (18), Chen and Zhang (19), Dilsen et al. (20), and Mizushima (21).

### The International Study Group for Behçet Disease criteria (1990)

The International Study Group for Behçet’s Disease (ISGBD) was assembled in London in 1985, during the third international conference on ABD with the purpose of reviewing the diagnostic criteria of ABD and create a new set of internationally approved criteria in order to conform and better compare the publications about ABD and to foster collaborations among different countries. More than 900 patients were recruited from 7 countries (Iran, Turkey, Japan, Tunisia, UK, USA, and France). The new criterion for the diagnosis of ABD was published in Lancet in 1990 and then validated in 1992 (22, 23).

According to the ISGBD, recurrent oral aphtosis (more than 3 episodes in a year) is mandatory for the diagnosis of ABD. A patient can be diagnosed with ABD when, in addition to oral ulcers, manifests at least two features among: genital ulcers, ocular lesions (anterior and posterior uveitis, retinal vasculitis), cutaneous lesions (pseudofolliculitis, erythema nodosum, papulo-pustular lesions, acneiform lesions), and positive pathergy test (22).

Pathergy test is a hypersensitive reaction that occurs 24–48 h after an intradermal injection of isotonic saline solution with a 20–22G needle on the forearm. The test is positive if a papule or a pustule with at least 2 mm diameter develops at the site of trauma (9).

### The limits of International Study Group for Behçet’s Disease criteria and the development of Iranian and Korean criteria

International Study Group for Behçet’s Disease criteria were created to guarantee uniformity in clinical studies rather than diagnose a single patient. Even if they were validated in different ethnical groups, a few items raised concern.

Firstly, since oral aphtosis was a mandatory feature, patients without oral ulcers (that represented 3% of 914 patients in the validation study) could not be diagnosed with ABD.

International Study Group for Behçet’s Disease recruited patients from countries in which the pathergy phenomenon has a high degree of positivity; therefore, the sensitivity of this test could be low in countries where the positivity is not as common. Patients with bipolar aphtosis (oral ad genital), before the arrival of ISGBD criteria, were considered to suffer from a mild form of ABD and, without a positive pathergy test, they could not meet the criteria for the diagnosis of ABD (24).

**FIGURE 2:**
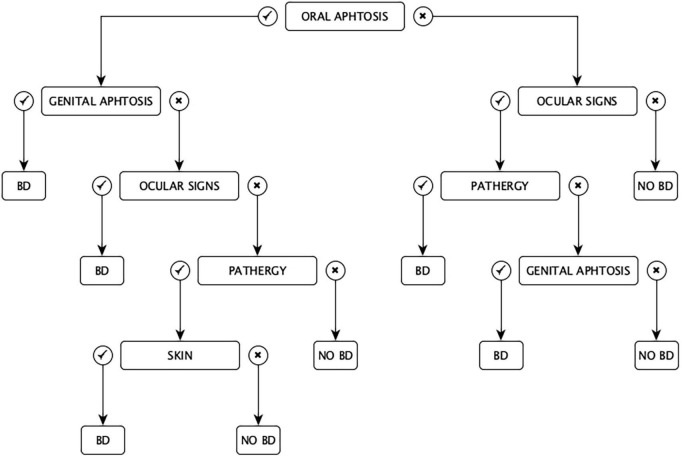
Iranian “classification tree” criteria for Adamantiades-Behçet disease.

In 1993, Davatchi and colleagues proposed the Iranian criteria for the diagnosis of ABD in a chart set. Having a “classification tree” set of criteria, according to the Authors, conferred a more appropriate weight to any symptom (Figure 2). In some cases, the diagnosis of ABD could be possible with only two features (e.g., oral aphtosis and genital aphtosis, oral aphtosis and ocular signs, ocular signs and pathergy, and genital aphtosis and ocular signs), in other cases, it could be established with at least three clinical manifestations (e.g., oral aphtosis plus ocular lesions and pathergy or oral aphtosis plus pathergy and skin manifestations) (25).

The main defect of this classification is the difficulty of the diagram because in many cases the scheme in order to follow the branches of the diagnostic algorithm should be kept in hand (26).

Korea did not take part in the study of ISGBD in 1990. In 2003, new guidelines developed by Korean authors were published to overcome the difficulties using ISGBD criteria in a country where only 35% of patients with ABD had a positive pathergy reaction. For the first time, numeric scores were assessed for every symptom: recurrent genital aphtosis scored 2 points, recurrent oral aphtosis scored 1 point, cutaneous lesions (erythema nodosum–like lesions, pseudofolliculitis, or papulo-pustular lesions) scored 1 point, ocular lesions (anterior/posterior uveitis, retinal vasculitis) scored 1 point, positive pathergy test scored 1 point, ileocecal ulcerations (excluding inflammatory bowel disease and intestinal tuberculosis) scored 1 point. ABD could be diagnosed with at least 3 points, and the presence of HLA-B51 could be useful for the diagnosis (27, 28).

### The International Team for the Revision of the International Criteria for Behçet’s Disease criteria

The International Team for the Revision of the International Criteria for Behçet’s Disease (ITR-ICBD) was created in 2004 not only to evaluate and uniform the existing diagnostic criteria for ABD but also to establish a new set of criteria with optimized sensitivity, specificity, and accuracy. Twenty-seven countries joined the study (Austria, Azerbaijan, China, Egypt, France, Germany, Greece, India, Iran, Iraq, Israel, Italy, Japan, Jordan, Liba, Morocco, Pakistan, Portugal, Russia, Saudi Arabia, Singapore, Spain, Taiwan, Thailand, Tunisia, Turkey, and USA) for a total of 3,719 patients. Also for ITR-ICBD criteria, a scoring system was created: oral aphtosis scored 1 point, cutaneous features scored 1 point, vascular lesions (arterial or venous thrombosis, aneurism) scored 1 point, positive pathergy test scored 1 point, genital aphtosis scored 2 points, and ocular lesions scored 2 points. ABD is diagnosed by reaching 3 or more points (29, 30). These new set of criteria appeared to have better sensitivity, specificity, and accuracy than ISGBD criteria in validation studies (31, 32).

### The new International Criteria for Behçets’s Disease

In 2014, the International Team for the Revision of the International Criteria for Behçet’s Disease (ITR-ICBD) published in *Journal of European Academy of Dermatology and Venereology* study about the performance of the new criteria. The study involved scientists from 32 countries.

According to the new proposed ICBD criteria, ABD can be diagnosed when a score of 4 or more points is reached. Ocular lesions, genital aphtosis, and oral aphtosis scored 2 points each; skin lesions, neurological manifestations, and vascular manifestations scored 1 point each. Pathergy test is optional, and when positive one extra point can be added (Table 1). Comprehending a wide number of features (with the addition of neurological manifestation), the new ICBD criteria should let physicians to diagnose early ABD in order to refer the patients to expert centers. Since pathergy test is no longer mandatory, these criteria can be used also in centers where pathergy is not a routinary test and in countries where the rate of positive pathergy test is low (33).

**TABLE 1:** The new International Criteria for Behçets’s Disease (ICBD) criteria for Adamatiades-Behçet disease.

Signs/Symptoms	Points
Oral aphtosis	2
Genital aphtosis	2
Ocular lesions	2
Skin lesions	1
Neurological manifestations	1
Vascular manifestations	1
Positive pathergy test (optional)	1

Adamantiades-Behçet disease is diagnosed with 4 or more points.

## Discussion

Due to the absence of a pathognomonic test, the diagnosis of ABD is clinical and in time a huge number of different diagnostic or classification criteria have been proposed by scientists. With the introduction of the new ICBD criteria, the collaboration among different specialties is mandatory for the diagnosis and the management of patients with ABD.

Within the diagnostic/classification criteria, a positive family history, the identification of HLA-B51 and the presence of articular manifestations and gastrointestinal signs, even if are often used for clinical diagnosis, have never been taken into account for the diagnosis of ABD.

With the passing of ISGBD criteria of 1990, patients without oral aphtosis showing other specific clinical features can be diagnosed with ABD and early treated.

## Author contributions

SB concepted the whole work and drafted and submitted the manuscript.
